# Spotted fever diagnosis using molecular methods

**DOI:** 10.1590/0037-8682-0226-2024

**Published:** 2024-11-15

**Authors:** Helen Gonçalves Marques, Anna Julia Ribeiro, Anna Karolina de Oliveira Alfenas Gadelha, Carlos Ananias Aparecido Resende, Daniela Regiane da Silva, Débora Patrícia Martins de Deus, Isabelle Caroline Dos Santos Barcelos, Isabela Maia Pereira, Iago Tadeu Santos de Paula, Lucas Da Silva Lopes, Líria Souza Silva, Mariana Campos da Paz Lopes, Miguel Angel Chávez-Fumagalli, Eduardo Antônio Ferraz Coelho, Rodolfo Cordeiro Giunchetti, Ana Alice Maia Gonçalves, Alexsandro Sobreira Galdino

**Affiliations:** 1 Universidade Federal de São João Del-Rei, Programas de Pós-graduação em Biotecnologia e Multicêntrico em Bioquímica e Biologia Molecular, Disciplina Biotecnologia & Inovações, Divinópolis, MG, Brasil.; 2 Universidade Federal de São João Del-Rei, Laboratório de Biotecnologia de Microrganismos, Instituto Nacional de Ciência e Tecnologia em Biotecnologia Industrial, Divinópolis, MG, Brasil;; 3 Universidade Federal de São João Del-Rei, Laboratório de Bioativos e Nanobiotecnologia, Divinópolis, MG, Brasil.; 4 Vicerrectorado Universidad Católica de Santa Maria, Computational Biology and Chemistry Research Group, Arequipa, Peru.; 5 Universidade Federal de Minas Gerais, Faculdade de Medicina, Programa de Pós-Graduação em Ciências da Saúde: Doenças Infecciosas e Medicina Tropical, Belo Horizonte, MG, Brasil.; 6 Instituto Nacional de Ciência e Tecnologia em Doenças Tropicais, Universidade Federal de Minas Gerais, Laboratório de Biologia das Interações Celulares, Belo Horizonte, MG, Brasil.

**Keywords:** Spotted fever, *Rickettsia* spp., Molecular diagnosis of spotted fever, Spotted fever group rickettsioses

## Abstract

Rickettsiosis is a disease caused by bacteria belonging to the genus *Rickettsia*, and is a potentially fatal zoonotic disease of great medical and veterinary importance. Given the urgent need to develop new diagnostic methods for detecting this disease, the present review aimed to evaluate the number of publications dedicated to the identification of *Rickettsia spp.* in human samples using molecular methods, such as polymerase chain reaction and its variations. To this end, a bibliographical survey covering articles published in the past ten years was conducted using the PudMed platform with the keywords “spotted fever” and “*Rickettsia*,” both combined with “diagnosis.” A growing number of publications in this area reflects an increasing interest in research, especially since 2015. From 2015 to February 2024, several promising results were tested and many studies were able to detect the genetic sequences of interest. Therefore, the absence of a standard diagnosis method highlights the critical need for developing an effective technique capable of accurately detecting the etiological agent and ensuring accurate diagnosis of the disease.

## INTRODUCTION

Rickettsiosis, a potentially life-threatening zoonotic disease of significant medical and veterinary importance, is caused by bacteria belonging to the genus *Rickettsia* ^1^. The bacteria of this genus are gram-negative, non-motile, obligate intracellular organisms that are transmitted by means of a vector such as lice, ticks, mites, and fleas. Transmission typically occurs either through bite or contact with the feces of infected arthropods^2^. More than 30 species comprise the spotted fever group rickettsioses (SFGR), with their respective diseases varying according to the regions where they occur^3^. The main bacteria that cause diseases in humans are *Rickettsia rickettsii, Rickettsia conorii, Rickettsia parkeri, Rickettsia africae, Rickettsia sibirica, Rickettsia slovaca, Rickettsia japonica,* and *Rickettsia honei* ^2^. Of these, the Brazilian spotted fever (BSF) and Rocky Mountain spotted fever (RMSF), caused by *R. rickettsii*, are considered the most severe^4^. 

The epidemiology of rickettsiosis is diverse and is closely related to the biology of the vector and its habitat. As the different hosts do not belong to the same species, the accurate identification of the disease is challenging^5^ ^,^ ^6^. Most cases of SFGR are geographically distributed across North America, Latin America, Southern Africa, Europe, the Mediterranean, and East Asia^7^. Between 2007 and 2021, 2,545 confirmed cases of SGFR were recorded in Brazil, with the southern and southeastern regions contributing to more than 80% of the cases. Furthermore, 834 (32.8 %) deaths occurred during the period^8^. However, many cases may be underreported because the disease can present with non-specific symptoms that hamper accurate reporting. This can result in erroneous diagnoses and treatment, which exacerbates disease severity due to delayed treatment ^9^ ^,^ ^10^. Symptoms typically include fever, headache, systemic rashes, discomfort, nausea, and even serious complications, such as vascular lesions, suspected necrosis, and gangrene^11^. Therefore, obtaining accurate diagnosis is essential. However, this can be challenging owing to the lack of adequate diagnosis and variations in epidemiological surveillance across different countries, complicating the reporting of the disease occurrences^12^.

Diagnosis of rickettsiosis can be based on different methods, such as the direct investigation of *Rickettsia* as well as molecular and serological methods, with the latter being the most commonly used^13^. However, serological tests are hindered by difficulties related to specificity and sensitivity, the risk of cross-reactivity with other diseases, and the requirement for multiple samples for accurate confirmation^14^ ^,^ ^15^. Thus, molecular tests offer promising alternatives for the diagnosis and epidemiological monitoring of *Rickettsia* infections because they are capable of detecting bacteria with a high degree of sensitivity and specificity^2^. 

Currently, polymerase chain reaction (PCR) is the most efficient molecular method available for investigating SGRF cases with high sensitivity and specificity^16^. This technique stands out for its versatility and is used for both qualitative and quantitative analyses in various diagnostic medical applications, such as the detection of microorganisms, viral load monitoring, and bacterial quantification^17^. Molecular methods are effective for diagnosing various diseases, including Corona virus disease 2019 (COVID-19), meningitis, hepatitis, tuberculosis, and acquired immunodeficiency syndrome (AIDS)^18^ ^,^ ^19^ ^,^ ^20^ ^,^ ^21^ ^,^ ^22^. In the case of *Rickettsia* infection, PCR allows the rapid detection of rickettsiosis DNA in different samples without the need for multiple tests with high sensitivity and specificity^23^. Despite its diagnostic efficacy, PCR presents with some challenges and limitations. One disadvantage is its susceptibility to contamination and the non-specific annealing of primers with similar DNA sequences, which can generate erroneous results^24^. Furthermore, the sensitivity and specificity of the PCR technique may be substantially reduced depending on the time of sample collection or the disease stage, which is also one of the main disadvantages of this method^22^. Moreover, sample type can affect diagnostic performance *Rickettsia* spp. multiply mainly inside endothelial cells, causing low levels of rickettsemia to be detected in circulating blood^25^ ^,^ ^26^. Given that a positive result is strongly correlated to endothelial damage^25^, PCR tests performed using blood samples tend to exhibit lower sensitivity. Considering the promising application of these methods and the need to develop more accurate methods for the diagnosis of rickettsiosis, this review aims to evaluate the effectiveness of molecular methods developed for the diagnosis of *Rickettsia* spp. in humans and to compile relevant information regarding the main techniques and their applications.

## MATERIALS AND METHODS

**FIGURE 1:**
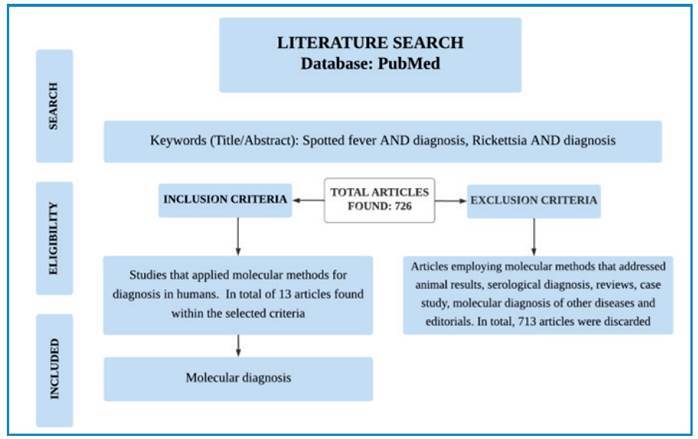
Flowchart illustrating the article selection process.

The PubMed database was searched for scientific articles. The following descriptors were used: spotted fever [Title/Abstract]) AND (diagnosis[Title/Abstract]) and *Rickettsia*[Title/Abstract]) AND (diagnosis[Title/Abstract]). A total of 726 articles were identified. All articles published in English over the past ten years (2013- Mar 2024) were included. Articles that focused on molecular methods involving animals, serological diagnoses, bibliographic reviews, case studies, epidemiological reviews, unrelated diagnoses, editorials, duplicate articles, and those unrelated to the topic were excluded. Only studies that used molecular diagnostics to detect human rickettsiosis were included. Figure 1 presents the article selection process.

## MOLECULAR DIAGNOSIS METHODS FOR SPOTTED FEVER GROUP RICKETTSIOSES

PCR technique was first described in 1984 by the biochemist Kary Mullis, and has since been widely used for the rapid and sensitive identification of various infectious and genetic diseases in animals and humans^27^ ^,^ ^28^. It stands out for its practicality, as the operation can be completed in a shorter time period compared to traditional culture-based identification^29^. Additionally, the analysis can be performed using small amounts of genetic material obtained from a wide variety of sample types^30^. Consequently, this method has been extensively studied and optimized, resulting in the development of several new techniques^31^.Of these, quantitative or real-time PCR (qPCR) operates on the same principle as conventional PCR, offering the advantage of immediate visualization of the results and eliminating the need for agarose gel electrophoresis^32^. This technique stands out for its high sensitivity and specificity in quantifying the abundance of specific genes/DNA sequences that can be monitored in each cycle^33^. Multiplex qPCR, another variation of this technique, allows the simultaneous detection and amplification of one or multiple DNA and RNA targets in a single reaction^34^. Moreover, nested PCR can reduce non-specific binding of primers^35^. In addition, other methods have also been developed, including semi-nested PCR, reverse transcription nested PCR (RT-nested PCR), and consensus-nested PCR.

Other molecular diagnostic techniques have also emerged as important alternatives for early and accurate disease detection^36^. The loop-mediated isothermal DNA amplification (LAMP) method, developed in Japan in 1998, allows the increase in the amount of amplified DNA, with enhanced assay sensitivity^37^ ^,^ ^38^. Recombinase polymerase amplification (RPA), another isothermal approach, amplifies target nucleic acids using recombinant enzymes at a constant temperature, offering a rapid and sensitive alternative for the molecular diagnosis of diseases^39^. Furthermore, the reverse line blot (RLB) method is another sensitive and specific alternative for the simultaneous detection of multiple pathogens^40^ ^.^ Despite the diversity of molecular methods that can be applied in disease diagnosis, only 13 studies in the literature have used molecular techniques to diagnose SFGR (Table 1 and Table 2), and their results are summarized below: 

**TABLE 1:** Primers and probes developed for the detection of SFGR infections.

Species	Gene-Target	Primers/ Probes	Reference
*Rickettsia* spp.	-	PanR8F-AGCTTGCTTTTGGATCATTTGG/PanR8R-TTCCTTGCCTTTTCATACATCTAGT	^39^
		PanR8P-Fl-CCTGCTTCTATTTGTCTTGCAGTAACACGCCA-BHQ1	
		RRi6F-AAATCAACGGAAGAGCAAAA/RRi6_R-CCCTCCACTACCTGCATCAT	
		RRi6P^c^-Fl-TCCTCTCCAATCAGCGATTC-BHQ1	
SFGR	16S rDNA *glt*A, *htr*A,	16S rDNA- fD1- AGAGTTTGATCCTGGCTCAG/ Rc16S.452n- AACGTCATTATCTTCCTTGC	^40^
	*omp*A and *ompB*	*glt*ARpCS.877pGGGGGCCTGCTCACGGCGG/RpCS.1258nATTGCAAAAAGTACAGTGAACA/RpCS.896p-GGCTAATGAAGCAGTGATAA/ RpCS.1233n- GCGACGGTATACCCATAGC	
		CS-78-GCAAGTATCGGTGAGGATGTAAT/CS323-CTTCCTTAAAATTCAATAAATCAGGAT	
		*htr*A-17 kDa-1- GCTCTTGCAACTTCTATGTT/ 17 kDa-2- CATTGTTCGTCAGGTTGGCA	
		*omp*A-Rr190.70p-TGGCGAATATTTCTCCAAAA/Rr190.701nGTTCCGTTAATGGCAGCATCT	
		Rr190.70p-ATGGCGAATATTTCTCCAAAA/ Rr190.602n-AGTGCAGCATTCGCTCCCCCT	
		*ompB-*rompBOF-GTAACCGGAAGTAATCGTTTCGTAA/rompBOR-GCTTTATAACCAGCTAAACCACC/ rompBSFGIF- GTTTAATACGTGCTGCTAACCAA/rompB SFG IR- GGTTTGGCCCATATACCATAA	
*R. rickettsii*	Hypothetical *R. rickettsii* protein gene, *gltA,* and *OmpB*	Rickettsial gltA gene-TCGCAAATGTTCACGGTACTTT/TCGTGCATTTCTTTCCATTGTG	^41^
*R. parkeri*		FAM-TGCAATAGCAAGAACCGTAGGCTGGATG-BHQ1	
*R. akari*		*R. rickettsii* hypothetical protein/AAATCAACGGAAGAGCAAAAC/CCCTCCACTACCTGCATCAT/CY5-TCCTCTCCAATCAGCGATTC-BHQ3	
		*R. parkeri ompB/* CAAATGTTGCAGTTCCTCTAAATG/AAAACAAACCGTTAAAACTACCG	
		FAM-CGCGAAATTAATACCCTTATGAGCAGCAGTCGCG-BHQ1	
		*R. akari ompB/*GTGGTGCTGTTGCAGGTGG/TTGCTCCACCGAGAGTTAATGTT	
		HEX-CGGTGCTGGTAATGCTGCATTACACG-BHQ1	
*R. japonica*	-	Ricko1-AGTAATGCACCTACACCTACTC/Ricko2-CGGGCGGTATGAATAAACAAG	^42^
*Rickettsia* spp.		PanRick/ RC0338	^43^
*Rickettsia* spp.	23S-5S rRNA	RLB-23S-5-F-GATAGGTCRGRTGTGGAAGCAC-3/RLB-23-5-R-TCGGGAYGGGATCGTGTGTTTC	^44^
*R. japonica*	16S rDNA	OR-F GGAGCATGCGGTTTAATTCG/OR-R GCCATGCAACACCTGTGTGT/(Ot-FAM) FAM-AATGGAGACATTTTTCTTC-MGB/ (Rj-VIC) VIC-CGGATCGCAGAGATG-MGB	^45^
*Rickettsia* spp.	MC1_07110	-	^46^
SFGR	*ompA*	F-TTGTCAGGCTCTGAAGCTAAAC/ R- AGCACCTGCCGTTGTGATATC	^47^
		P-TAGCCGCAGTCCCTACAACACCGC-BHQ1	
*Rickettsia* spp.	23S-5S rRNA	GGAAGCACAGTAATGTGTGTAG/TCGGGAYGGGATCGTGTGTTTC	^48^
		P-TAGCTCGATTGRTTTACTTT	
*Rickettsia* spp.	23S rDNA, and rRNA	F-GGTCCCACAGACTTACCAAACTCA/ R-TCGACTATGGACCTTAGCACCCAT P-FI/CCGAATGTCGATGAGTACAGCATAGCAGAC-BHQ1	^20^
*Rickettsia* spp.	17-kDa	RPA17F/AATTCACAACTTGCCATTGTCCGTCAGGTT/RPA17R/RBiotinCTGTAACGGTCCAGGCGGTATGAATAAACA/	^7^
		RPA17P/FAM-CCGGATTACGCCATTCTACGTTACTACCAC[THF]	
		RPA47F/GTATCAACAAGAAGTGTTCTTAGGTTCTGG/RPA47R/BiotinCCTTCAAGATTAAACATCGGTCCACCAAAG/AGGAGCTGTTTCTAAAG-C3Spacer/	
		RPA/47P/Digoxin/CATGATGGTTCAGAACTGATAGCAGAATTA[THF]TTGGCAGTGACAATA-C3Spacer/snRPA17F/TTATTAGGTGTTACGTAACCGTAATTGC/snRPA47F/GAGAATGTTATAGCGGGAGCTGAAAAT	
		ATA/TaqMan17F/ACAGGATAGAAGACTTGCAGAGCTTAC/TaqMan17R/CAACTTGCCATTGTCCGTCAGG/TaqMan	
		47F/CTGTACTTGAAGCAGTTGAATGC/TaqMan47R/TTGTGCTGCAGATCCTTCTT/TaqMan17P/FAM/ACAGCTCCTA	
		GTGGTAGTAACGTAGAATGG-BHQ1/TaqMan47P/FAM-TCATTAAGCATAACATTTAACATACCACGACGA-BHQ1	
*R. rickettsii*		FIPRrickettsii/CGCTGTGGATTAAAGGAGAAGGTCAAGttttCTATAGATGCTAGTGCAGAAATAGCAACAACTC/BIPRrickettsii/GGCAAGTGTTGTCATTAACCCTTTTCCTTTTCCttttGGCCAGACTTTGCCGATTTCAAGG/F3Rrickettsii/GGCTTT	^49^
		AATTGTAGCGGGGGAATGAATAGA/B3Rrickettsii/GCATCGCAATTAAAGCTGCATCAGCTAA/LPRrickettsii/CCTTTACCTCTCCTGATTCTCACTTTGTGGGC/LFRrickettsii/CGGCTATGCTCCAGCTCACCAT	

**SFGR:** Spotted fever group rickettsioses; **JSF:** Japanese spotted fever; **TNA:** Total nucleic acid; **RNA:** ribonucleic acid; **DNA:** deoxyribonucleic acid; ***glt*A:** citrate synthase gene; ***htr*A:** 17 kDa protein; ***omp*B:** outer membrane protein B**; *omp*A:** outer membrane protein A.

**TABLE 2:** Sample types and results.

Sample	Test	Results	Reference
27 blood samples and 14 tissue biopsy samples	Real-time PCR	PanR8 - blood and tissue biopsy: 100% positive RRi6 - blood samples: 23/27 positive; tissue biopsy: 5/14 positive	^39^
16 blood samples treated with EDTA, 2 plasma samples, 4 buffy coat samples, 12 samples of erythrocyte portions of the samples, 8 serum samples, and 3 skin biopsy samples	Single and sequential PCR	Single-stage PCR - Buffy coat: 25% (16S rDNA); skin biopsies: 33% (*glt*A); erythrocyte portion: 6.25% (*ompB*); EDTA Blood 6.25% (*ompA*) was positive	^40^
		Nested or semi-nested PCR - Buffy coat: 25% (*gltA*) and 100% (*ompB*); skin biopsies:100%(*gltA)*; 66.6% (*ompA*) and 100% (*ompB*); EDTA blood: 50% (gltA), 25% (*ompA*) and 75% (*ompB*); erythrocyte portion: 50% (*gltA*), 16.6% (*ompA*) and 83.3% (*ompB*); Plasma: 100% (*gltA* and *ompB)*, Sera: 50%, 12.5% and 62.5% were positive	
		No samples were positive using the *htr*A gene	
31 positive skin biopsy samples	Multiplex qPCR	Multiplex Qpcr - correctly determined the presence of *R. rickettsii*, *R. parkeri,* and *R. akari* in the samples	^41^
Plasma, peripheral blood mononuclear cells (PMBC), erythrocyte and neutrophil fractions from 34 blood samples positive for JSF	Conventional PCR and Rick PCR (nested)	Nested PCR - 17.6% (6/34) without plasma; 23.5% (18/34) in PBMC and 20.6% (7/34) in erythrocyte and neutrophil fractions were positive	^42^
		Rick PCR - 76.5% (36/34) in plasma; 73.5% (25/34) in PBMC, and 55.9% (19/34) in erythrocyte and neutrophil fractions were positive	
44 skin biopsy samples and 79 whole blood samples	qPCR	Skin biopsies: PanRick: 24 (54.5%); RC00338: 19 (43.2%)	^43^
		Whole blood samples: PanRick: 5 (6.3%); RC00338: 3 (3.8%)	
69 skin biopsy samples, 15 cutaneous swab samples, and 37 cerebrospinal fluid samples	qPCR and RLB	RLB - skin biopsy samples: 40.6%; swabs: 46.7%	^44^
		qPCR - skin samples: 63.7%; swabs: 80%	
		Compared to qPCR, RLB had 46.4% sensitivity	
33 samples from Akita individuals, 67 samples from Fukushima individuals, 28 samples from Shizuoka individuals, 22 samples from Wakayama individuals, 28 samples from Miyazaki individuals, and 139 samples from Hiroshima patients	Real-time PCR	Akita: 0/33; Fukushima: 0/67; Shizuoka: 11%; Wakayama: 41%; Miyazaki: 25%; Hiroshima: 40%	^45^
175 blood samples from the Medical College of Wisconsin and 250 blood samples from the Gunderson Medical Foundation	HDPCR multiplex	Sensitivity: 100%; Specificity: 97.3%	^46^
	TBP		
20 SFGR positive blood samples	qPCR Multiplex	Sensitivity: 67%; Specificity: 95%	^47^
89 fresh tissue samples,12 lymph node samples, 17 exudate samples, 39 cerebrospinal fluid samples, 55 blood samples	PCR-RLB	Sensitivity: 99.5%; Specificity: 100%	^48^
12 serum samples, 19 blood samples, 3 cerebrospinal fluid samples, 4 Swab samples, 3 plasma samples, 1 sample of spleen, kidney, lung, liver, skin, and brain.	rtRT-PCR	6 serum samples, 5 blood samples, and others, such as plasma, cerebrospinal fluid, spleen, kidney, lung, liver, skin, and brain samples, were positive for *R. rickettsii*	^20^
642 blood samples collected from Yunnan, 236 blood samples collected from Inner Mongolia	snRPA-nfo and TaqMan PCR	*R. rickettsii*, *R. sibirica*, *R. japonica* and	^7^
		*R. montana* were positive for RPA-nfo-17 and TaqMan-17	
50 positive human samples	LAMP	LAMP-HNB- Sensitivity: 93%; Specificity: 70%	^49^
53 negative human samples		LAMP-ELECTROPHORESIS- Sensitivity: 97%; Specificity: 58%	

**SFGR:** Spotted fever group rickettsioses; **JSF:** Japanese spotted fever; **TNA:** Total nucleic acid; PCR: polymerase chain reaction; **HDPCR:** high-definition polymerase chain reaction; **TPB:** tick-borne panel; **LAMP:** loop-mediated isothermal DNA amplification; **qPCR:** quantitative or real time PCR; **snRPA-nfo:** polymerase amplification assay; **rtRT-PCR:** reverse transcriptase PCR; **RLB:** reverse line blot; **PanR8:** Pan-*Rickettsia* real time PCR assay, and **RRi6:** *R. rickettsii* real time PCR assay.

Kato *et al*. (2013)^41^ developed two real-time PCR assays to detect *Rickettsia* spp., with one being specific for *R. rickettsii*. To perform the assay, the primers PanR8_F, PanR8_R, and PanR8_P were used for the Pan-*Rickettsia* real time PCR assay (PanR8), and the primers RRi6_F, RRi6_R, and RRi6_P were used for the *R. rickettsii* real time PCR assay (RRi6). In total, 41 positive blood and tissue biopsy samples, previously diagnosed by 17kDa nested PCR, were used. Among these samples, 20 were from fatal SFGR cases, four from non-fatal cases, and one was of unknown outcome. In addition, three tissue biopsy samples were from fatal cases, nine were from non-fatal cases, and two had undetermined outcomes. The PanR8 assay detected all the samples tested. The RRi6 assay detected all the fatal blood samples, two non-fatal samples, and one unknown sample. Of the tissue biopsy samples, RRi6 detected two fatal, three non-fatal, and one unknown case.

Subsequently, Santibáñez *et al.* (2013)^42^ evaluated the sensitivity of PCR assays for identifying *Rickettsia* species. Primers were designed to target the 16S rDNA, citrate synthase gene (*glt*A), and 17 kDa protein (*htr*A) genes for the three single-stage PCR assays, and the outer membrane protein B (*omp*B), *glt*A, and outer membrane protein A (*omp*A) genes for the three nested or semi-nested PCR assays. A total of 72 clinical samples of ethylenediaminetetraacetic acid (EDTA)-treated blood, plasma, buffy coat, erythrocyte portions of specimens, and sera and skin biopsies were collected from 52 individuals with PCR-diagnosed rickettsiosis. In single-stage PCR assays, 25% of buffy coat samples were detected using 16S rDNA, 33.3% of skin biopsy samples using *glt*A (5'e), and 6.25% of blood EDTA using *omp*A PCR tests. Sequential PCRs detected rickettsial DNA in 100% of skin and plasma biopsy samples for *glt*A and/or *omp*B. Furthermore, all the buffy coat samples tested positive for *omp*B, 25% for *omp*A, and 100% for *omp*B. Regarding the erythrocyte portion samples, 83.3% were positive for *omp*B, 16.6% were positive for *omp*A, and 50% were positive for *glt*A. Among the EDTA blood samples, 75% were positive for *omp*B, 50% for *omp*A, and 25% for *omp*B. For the serum samples, 50% were positive for *glt*A, 12.5% for *omp*A, and 62.5% for *omp*B. A sensitivity of 100% was achieved when three sequential assays were performed.

Denisson et al. (2014)^43^ evaluated a multiplex real-time PCR assay for detecting *Rickettsia* spp. Primers targeting *glt*A, the *omp*B genes from *R. parkeri* and *R. akari* and a hypothetical protein gene from *R. rickettsii* were used. To evaluate specificity, 10 species of *Rickettsia* were cultivated in Vero E6 cells and prepared to control the assay. A total of 72 clinical samples of EDTA-treated blood, plasma, buffy coat, erythrocyte portions, serum, and skin biopsies were collected from 52 individuals with rickettsiosis diagnosed using PCR. Among the samples, the authors included both confirmed and suspected cases of SFGR tested by culture or nested PCR for *ompA* in specific species. Of these, three samples were confirmed cases, one was suspected of RMSF, six were confirmed and three were suspected of *R. parkeri* rickettsiosis, two were confirmed and 11 were suspected of Rickettsialpox, four were confirmed for African tick bite fever, and one was confirmed for *Rickettsia* 364D rickettsiosis. All samples confirmed and suspected to be RMSF tested positive using *glt*A and *R. rickettsii* hypothetical protein genes. All confirmed and suspected *R. parkeri* rickettsiosis samples were positive for *glt*A and *R. parkeri omp*B genes. Of the samples confirmed and suspected of Rickettsialpox, all were positive for *glt*A and *R. akari omp*B. Moreover, all confirmed and suspected samples of African tick-bite fever and *Rickettsia* 364D rickettsiosis were positive for *glt*A.

Kondo *et al.* (2015)^44^ developed a molecular diagnostic technique for detecting *R. japonica*. To conduct this study, the authors developed Rick PCR using the *R. japonica* 17 K genus common antigen gene. A new set of primers, Ricko1 and Ricko2, were tested using Rick PCR. This study included 34 samples obtained from infected and clinically diagnosed individuals, where the blood fractions were evaluated separately. Nested PCR identified 31.0% (9/29) positive whole blood samples, 17.6% (6/34) positive plasma samples, 23.5% (8/34) positive peripheral blood mononuclear cell samples, 20.6% (7/37) positive erythrocyte and neutrophil samples, and 28.6% (4/14) positive serum samples. Despite these results, compared to conventional nested PCR, Rick PCR demonstrated increased sensitivity. 

Znazen *et al.* (2015)^45^ tested two qPCR methods to detect *Rickettsia* spp. For the first assay, the PanRick primer set was used to target all *Rickettsia* species. The second qPCR, named RC0338, detected all SFGR samples. Samples from 123 individuals with previously confirmed SFGR infection via serological diagnoses were used, including 44 skin samples, where PanRick detected 54.5% of the samples (24/44), whereas RC00338 detected 43.2% (19/44). Among the 79 whole blood samples, 6.3% (5/79) and 3.8% (3/79) were positive for PanRick and RC00338, respectively. 

Khrouf *et al.* (2016)^46^ evaluated the diagnostic accuracy of qPCR and RLB in detecting *Rickettsia* spp. For qPCR amplification, *R. conorii, R. felis,* and *R. africae* specific probes and RC00338 primer were used. To test the RLB hybridization assay, primers targeting the 23S-5S rRNA gene region were used. In total, 121 skin samples, skin swabs, and cerebrospinal fluid samples from 101 symptomatic adults with clinically suspected rickettsiosis were used in this study. The RLB assay detected the presence of *Rickettsia* DNA in 40.6% of skin biopsy samples and 46.7% of swabs, whereas qPCR identified infections in 63.7% of skin samples and 80% of swabs. *R. conorii* was the most commonly identified species in both assays. An agreement of 68.6% was observed between the two techniques. 

Kawamori *et al*. (2018)^47^ developed a new diagnostic assay for SFGR detection, using a, real-time duplex PCR. A new set of primers, named Rj-VIC, was later compared with conventional PCR. The primers used in this study were designed based on the conserved SFGR sequences. A total of 317 samples from individuals with clinically suspected or confirmed rickettsiosis in hospitals from 1997 to 2016 across different regions of Japan were analyzed. A total of 74/317 samples from different endemic regions of Japan were positive. In the assay, the following *Rickettsiae* were positive for Rj-VIC: *R. japonica*, *R. heilongjiangensis*, *R. asiatica*, *R. conorii*, *R. helvetica*, *R. honei*, *R. rickettsii*, *R. sibirica*, *R. tamurae*, *R. australis*, *Rickettsia spp.*, *R. monacensis,* and *R. prowazekii*. When analyzing sensitivity, in comparison with conventional PCR, both methods detected approximately the same number of copies.

Buchan et al. (2019)^48^ evaluated the performance of a new PCR assay for the detection of tick-borne infections, including *Rickettsia* spp. High-definition PCR (HDPCR) and Tick-Borne Panel (TBP) assays were used, and the results were compared with those of a traditional multiplexed PCR test. For HDPCR/TBP, primers were designed to identify multiple pathogens, including a primer targeting the MC1_07110 gene, corresponding to the *Rickettsia* 17kDa protein antigen. In total, 425 blood samples collected from individuals suspected of having tick-borne infections were evaluated, as well as 93 synthetic samples that included single- and dual-target specimens. A tick-borne pathogen was identified by HDPCR, TBP, or reference PCR in 27/425 (6.4%) samples analyzed. TBP identified pathogen in of the 20/425 (4.7 %) samples, including *Rickettsia* spp. (n=2). The reference test identified a pathogen in 23/425 (5.4%) samples, including *Rickettsia*spp. (n = 1). When evaluating the performance of the tick-borne HDPCR panel among synthetic samples, the results demonstrated 100% sensitivity and 97.3% specificity for *R. rickettsii* identification.

Reller and Dumler (2020)^49^ developed a multiplex qPCR assay to aid in the diagnosis of SFGR, and the results were compared with those of a singleplex qPCR assay. The gene target used in their study was the SFGR *consensus ompA* sequence, and the primers were designed using AlleleID 6 (Premier Biosoft, Palo Alto, CA) software. The samples included the blood, buffy coat, and blood DNA samples from 20 infected individuals with positive SFGR cases confirmed by serological testing, PCR, culture, or observation of morulae in blood leukocytes, in addition to 20 negative controls. The assay achieved a sensitivity of 67% and a specificity of 95%. No differences in detection sensitivity were observed between singleplex and multiplex qPCR assays.

Jado et al. (2020)^50^ developed a new diagnostic kit, a tick-borne bacteria flow chip (TBFC), to detect *Rickettsia,* among other agents, and compared the results with those of the PCR-RLB assay. Primers targeting the 23S-5S rRNA intergenic spacer were developed. A total of 212 serum samples from fresh tissues, lymph nodes, exudates, cerebrospinal fluid, and blood samples from suspected individuals with different symptoms and clinical signs consistent with multisystem disorders were used. The results indicated high agreement (99.53 %) between the TBFC Kit results and RLB. The number of positive samples in both tests was 21 (9.9%) for fresh tissue, 1 (0.5%) for cerebrospinal fluid, and 5 (2.3%) for blood. Of the remaining samples, 184 tested negative for *Rickettsia*. Furthermore, the results of only one lymph node aspirate sample showed discrepancy between the assays; it was negative in PCR-RLB and positive in TBFC. The TBFC demonstrated 99.5% sensitivity and 100% specificity for detecting *Rickettsia* infections.

Subsequently, Chung et al. (2022)^22^ described a real-time reverse transcriptase PCR (rtRT-PCR) assay, called RCKr for detecting *Rickettsia* spp. and compared the results with those of PCR performed in a previous study (PanR8)^41^. The RCKr assay target genes included 23S rDNA and rRNA. In this study, 49 samples with infections confirmed by real-time PCR or sequencing were used, which included samples from the blood, plasma, serum, swab, eschar, spleen, kidney, lung, liver, skin, brain, and cerebrospinal fluid. Among the samples, 18 samples were from eight fatal cases of RMSF, five samples from four different non-fatal RMSF cases, and one sample from a case with an unknown outcome. Additionally, nine samples were from indeterminate rickettsiosis cases, which included three non-fatal cases and four with unknown outcomes. Moreover, ten non-rickettsiosis samples, which included seven non-fatal cases and three with unknown outcomes, were also included. The RCKr detected *R*. *rickettsii* DNA in 18 clinical samples from eight fatal cases of RMSF, three samples from non-fatal cases, and one sample from unconfirmed rickettsioses with unknown RMSF outcomes. In samples from individuals with undetermined rickettsiosis, RCKr was detected in five samples derived from four infected individuals. Overall, RCKr was able to successfully detect all previously positive samples, along with two other samples that PanR8 failed to detect. Moreover, RCKr showed a 100-fold increase in CT values compared to PanR8. 

Zhang et al. (2023)^7^ described the development of a novel semi-nested recombinase polymerase amplification assay (snRPA-nfo) to detect and distinguish pathogenic *Rickettsia* species in human samples and compared it with TaqMan PCR. Forward and reverse primers and probes targeting the 17kDa protein genes of *R. sibirica* were designed. To validate the assay, 16 Taqman PCR-positive (ct≤35), 5 indeterminate (35 < Ct < 40) and 4 negative (Ct ≥ 40) samples from a previous study were used^51^. The results of the RPA-nfo assays demonstrated that, out of the 16 samples that were tested positive using TaqMan, 14 also tested positive using the snRPA-nfo method. Among the indeterminate samples, one tested positive in the snRPA-nfo assay. Notably, none of the negative samples tested positive using the snRPA-nfo assay. When compared to the positive results of TaqMan PCR, the snRPA-nfo assay showed a positive detection rate of 77.78%, in addition to 12.5% positive identification of previously indeterminate samples, demonstrating an enhanced detection rate compared to TaqMan PCR.

Carvajal-Gamez et al. (2024)^52^ developed a LAMP, using hydroxy naphthol blue (HNB) (LAMP-HNB), for the detection of *R. rickettsii* DNA, and evaluated its diagnostic usefulness by comparing the results with a PCR protocol previously described by Eremeeva et al. (2003)^53^. A set of six primers, FIPRrickettsii, BIPR*rickettsii*, F3R*rickettsii*, B3R*rickettsii*, LPR*rickettsii*, and LFR*rickettsii*, targeting the putative *R. rickettsii* gene, were used in this study. To evaluate this technique, 103 samples were used, of which 30 and 73 were confirmed as negative and positive for *R. rickettsii*, respectively, by PCR. The LAMP-HNB technique showed 93% sensitivity and 70% specificity.

## DISCUSSION

Despite advances in technology for improving diagnostic techniques, diagnosing rickettsiosis continues to be a challenge. In fact, clinical and laboratory diagnoses face bottlenecks because the low specificity of initial symptoms is often confused with other less fatal diseases^28^. In addition, serological diagnosis, the most routinely employed test in laboratories, generally has a low sensitivity, especially at disease onset^45^. Therefore, it is essential to develop and improve diagnostic methods to enable the accurate identification of rickettsiosis and improve the chances of survival. Owing to its high specificity and sensitivity in diagnosing the disease, PCR is a promising alternative, as it focuses on a small segment of DNA and is considered the gold standard in the diagnosis of numerous diseases^54^.

Recently, researchers have sought to develop sensitive and viable molecular tests for use in locations where rickettsiosis is present. However, the sensitivity varies according to the quantity of target DNA present in the samples. In cases of more recent infections, the amount of available DNA is often lower than during the late stages^44^. When developing a PCR assay, it is important to target stably expressed genes for the test as this is essential for accurate PCR results^55^. Another crucial point is the design of primers and probes, as they are fundamental to the success and quality of the analysis, because accurate and reliable quantification depends on their effective^56^.

Given the need to improve the diagnosis of rickettsiosis, variations in PCR have been developed, such as qPCR and multiplex HDPCR, in addition to other techniques, such as TBFC and LAMP. These techniques have demonstrated promising results in detecting the presence of the pathogen. Most studies that calculated the sensitivity and specificity of the developed assay showed good results in detecting *Rickettsia* species. In relation to the results described above, among the developed tests, the TBFC proposed by Jado et al. (2020)^50^ presented better results in terms of specificity and sensitivity values, as it was a faster and fully automated alternative compared to previous techniques, in addition to being sensitive and specific to the different samples being tested, including body fluids or biopsies. Nonetheless, it is not possible to infer that this would be the best test for routine use, given that all the aforementioned studies used different sample types, making performance and cost-effectiveness comparisons in clinical environments challenging. The genes encoding *htr*A, *gtl*A, *omp*B, *omp*A, and 16S rRNA have been targeted for primer design in many studies, demonstrating that they are crucial genes for the identification of *Rickettsia* species. Notably, these genes are found in SFGR and are capable of confirming the presence of the pathogen, depending on the primers used^57^. Although the 16S rRNA gene is known to exclusively characterize the sequences of all *Rickettsia* species^58^, the use of multiple gene targets is strongly recommended because it increases the chances of ensuring accurate identification^2^. Therefore, studies using multiple gene targets represent a promising alternative for the accurate diagnosis of infection by *Rickettsia* spp.

Despite the encouraging results of the aforementioned studies, some negative aspects must be highlighted. For example, some studies used samples from individuals with clinical suspicion of the disease to evaluate the performance of the technique. This is not ideal because it can directly influence the results, preventing a proper evaluation of the primer used. To obtain a better evaluation of the test and primer’s performance, properly characterized samples, both negative and positive, must be used. Additionally, the primers used in most studies were not specific to a particular type of *Rickettsia* but rather to SFGR, which could compromise the evaluation of the test’s specificity. Furthermore, several studies had relatively small sample sizes, making it necessary to evaluate different protocols using a larger sample size for a broader view and more reliable results. Therefore, tools that calculate the samples to be tested should be taken into account^59^. 

In summary, the use of molecular methods for the diagnosis of rickettsiosis, especially at the onset of the disease, is a useful tool, as erroneous diagnoses might occur based on negative serological results, which could delay treatment and increase the potential for pathogen spread. However, further studies in this regard are required, especially, multicenter studies to validate the best diagnostic method for rickettsiosis and standardize a protocol that meets the specificity and sensitivity criteria. Considering the worldwide prevalence of the disease, especially in developing countries, these methods should be cost-effective to ensure proper diagnosis and treatment of low-income populations before the disease becomes fatal.
